# Her Heart's Mistake

**Published:** 1889-06-08

**Authors:** E. D. Cumming

**Affiliations:** Author of "An Ordeal by Silence"


### Her Heart's Mistake.

By E. D. Cumming, Author of "An Ordeal by Silence."

*(Continued from page 141.)*

### Chapter IX.—A Parting.

The Medical Board, which had been summoned to "sit upon" George Bairdsley, had assembled, discussed his case, refreshed itself with iced pegs, and given its decision. The patient was ordered home on six months sick leave, and told that he would be sent off as soon as he was strong enough to be moved. But, to the breathless astonishment of all the three doctors who constituted the "board," he fought against their decision, and begged them to send him to the hills. Such a thing was absolutely unheard of in their experience, and they wondered what could possess the man who thus avowed his preference for Simla over England. Evidently he had not fully considered their verdict, so they consented to delay sending in their official report until the following day to give him time to recover his reason, as Dr. Anson, the president, remarked.

The fact was that Bairdsley shrank from the idea of parting with Kate, and worried himself over the thought of going home to such an extent that Dr. Harrison, who had not officiated on the board, felt that it would be necessary to let him have his own way. If he continued to fret as he was doing now, he would be down with fever again. So he undertook to interview Dr. Anson and the others, and persuade them to change their recommendation from leave to England to leave to Simla. He succeeded in doing this without difficulty, and hastened home from the club, where he had seen the members of the board, to set the patient's mind at rest.

Bairdsley was terribly weak and pulled down—a mere shadow of his old self—his arms and limbs were scarcely recognisable to himself, and he could not walk across his room without assistance, but now he had shaken off the fever his spirits had revived, and he felt that he had only to eat to recover completely. His satisfaction was only equalled by Kate's, when they learned the result of Dr. Harrison's representations. Simla was distant enough, but the mails only took about eighteen hours to travel between that station and Meerut, so correspondence could be easily maintained.

The longed-for monsoon burst that evening, and the fresh earthy scent and the general cleanliness which soon possessed the world without made Meerut a new place altogether. The fall in the temperature raised everyone's spirits, and even the horses might be heard neighing with delight in the stables.

"It makes me long to go out and make mud-pies," said Captain Phelps to Kate, as he came out on to the verandah, after paying his usual visit to George Bairdsley. "Umbrella? No thanks, Miss Harrison. I'm going to walk down to the club for the pure pleasure of getting wet through."

And the Captain betook himself to the club, where he had two extra pegs to celebrate the advent of the rains.

"One really ought not to want drinks in this weather," he remarked apologetically; "but the monsoon, like Christmas, breaks but once a year, and deserves notice if only on that account. Thanks, Wilton, I think I *could* do just half a brandy cocktail, since the evening is so damp."

Steadily, heavily poured the rain all that night, with a dull hissing roar which gladdened the hearts of men, and kept them awake listening to its music. The water gushed in thick streams from the angles of the roofs, first brown and turbid, then clear and sparkling as a mountain brook; it fell in heavy fringes from the eaves, hollowing gutters for itself in the thirsty ground below. It rained as though the clouds knew they had been late in coming, and wished to make up for their delay; rained in a hearty liberal fashion as it never rains at home. There is something in its very suddenness that makes the first monsoon shower a doubly welcomed blessing. For a few hours dark, ominous cloud-banks have obscured the south-western horizon, and loom larger and blacker as the eye watches; nearer they come and darker, while cattle bellow and crows croak and flap their dusty wings in welcome. It has come! the sun's glowing face is veiled, and the living world pants in its eagerness. One heavy drop raises a tiny puff of dust upon the road. Another and another, and in a single moment more heaven's floodgates fly open, and the monsoon has burst in all its blessed fury, calling forth the flower and leaf, which seemed to have lain all prepared to obey its bidding. There is no such sight for eyes worn red by the scorching sun as the bursting of the monsoon.

George Bairdsley made rapid progress now the hot weather was past, and a few days after his board had sent in their report and obtained his leave, he began his preparations for flight to Simla. He selected that in preference to any other station because he had friends there; Mussoorie was more easily reached from Meerut, but he knew no one at that place, and he wished to see the famous hill capital.

"I wish you could come with me, Kitty," he said for the twentieth time, as he sat with her in the verandah watching the rain. He was able to crawl about the house now without assistance, and had been allowed to take one short drive in the landau, which, however, proved too much for him.

"If I went Aunt Agnes would, of course, have to come too, George. I should like to go, I need hardly say, but I can't leave papa for the last few months of his stay in the country."

"I wish he could have retired now; we might all have gone home together then."

"You have never told me about your home, George; I only know that it's in Surrey. Tell me all about it now."

And Bairdsley, nothing loth, described the English home where he had come into the world, and passed his boyhood. It was an old ivy-clad manor, dating from Elizabeth's time, nestling among the Surrey hills, sheltered by oaks and elms older than the ancient building itself. A large rambling house, with deep, diamond-paned windows, which his father would not replace by more modern casements, though they kept out half the daylight that struggled through the woods around in the short winter days. It was a show-place in the summer, for the gardens were as old fashioned as the house. George Bairdsley lay back in his chair as he painted the lawn sloping down to the river, bounded by beds a mass of brilliant colouring, and orchards the envy of the country side, with an enthusiasm that made his listener's heart beat, while she wondered how he ever came to leave an inheritance so fair for life in the East.

"We used to hear such tales of Indian fruit," he said, "the pines, and the mangoes, and the leeches, and I don't know what all. Why, no Indian fruit that ever grew is worth one English strawberry or plebian gooseberry!"

"Indeed, you are right, George," sighed Kate, whose home life, with a querulous and stingy grandmother, had not left too many tender memories behind. "I was positively wild with delight when I was sent for by papa, and I must say I have enjoyed Indian life; but I'm not surprised at your regretting the change."

"I don't regret it," said Bairdsley, glancing at her.

"Still, George, I can't help comparing your English home with this; it doesn't look quite so bad now the rain has come, but it's a wretched country. I wonder you did not accept the doctor's offer and go home."

"And leave you?"

A little "passage of arms" followed the query, which requires no comment.

"What is your father like, George? Is your mother alive?" enquired Kate after a time.

"The governor is alive, but was not in very good health when I left. I am a bad correspondent, and, as the property is entailed, I don't bother much about writing."

"Are you the only son?" she enquired gravely. That remark about the entailed property sounded horrible to her. Did he mean that he would have behaved more dutifully to his father had his prospective interests recommended it? Her own father was the one idol of her life until she set George Bairdsley up beside him, and to hear him speak thus of his parent shocked her beyond measure.

"Yes, I'm the only son—only child, for that matter. My mother died years ago. I can scarcely remember her."

"Then does your father live all alone?"

"Yes, with a half-dozen old servants."

"He must be very lonely, George."

"I daresay he finds it slow. The Manor is the most melancholy place to live at you can well imagine. I never spent more than a week there after I joined, unless business connected with the estate required it. I was simply bored to death, and preferred town. But we will make The Manor a trifle livelier some day, Kitty; you and I."

"I should like to be able to brighten up your father in his lonely old age," said the girl, thoughtfully.

"I'm afraid you will never have a chance. He was growing very feeble when I last saw him, a fortnight before I left home; and I should never be surprised to hear that he had joined the majority."

"I wish you wouldn't speak of your father in that way, dear. I don't like it."

He laughed carelessly, unconscious that his mirth pained her more deeply than his words.

"One's parents must go, Kitty; Providence reconciles us to the inevitable."

"Your submission to the decrees of Providence is too cheerful to please me, George. I never expected to hear *you* speak like this."

Bairdsley looked at her, and the clouded brow warned him to alter his strain.

"I am sorry I hurt you, Kitty. Perhaps I do speak too lightly of these things; more lightly than I mean to do. I have never failed in my duty to the old man, and we get on exceedingly well together."

"Have you written to him since you came out?"

"Yes; I wrote once to tell him I'd arrived here."

"Doesn't he write to you?"

"Oh dear, yes; every mail. Poor old man, it must be an undertaking for him to make out a letter, with his failing sight and shaky hand."

"You have not written to tell him of our engagement yet, I suppose?"

"No; but really, Kitty, I had hardly time between the day it was settled and the day I came to grief."

"There were only four days; but I thought you might have considered that the matter would be sufficiently interesting to him to send him a line."

"Oh, he will be delighted; he was always urging me to find a wife and settle down at The Manor."

"George, will you promise me something?"

"Yes, anything—everything."

"Make a point of writing to your father next mail ——"

"I will."

"And every mail afterwards."

"Oh, my dear Kitty!"

"And every mail afterwards," repeated Kate with emphasis, "if it's only half-a-dozen lines."

"Won't once a month do?"

"No, certainly not; but you needn't do it at all if you don't care to please me."

"I'll do it willingly if it will give you any gratification."

"It will, a great deal, though I wish you would do it without that incentive."

"The governor will think I am feeling my way to asking for an increased allowance."

"You can easily disabuse him of that idea, if there's the least likelihood of your dutiful conduct giving him such a notion, by not asking anything of the kind."

"I'm not going to; he gives me more than I want, and it won't be very long now before I come into everything."

He spoke with the careless satisfaction which had roused Kate's ire before, and she bit her lips with disappointment and vexation. George Bairdsley was showing her a new side of his character, and one of whose existence she had never dreamed. How could he suppose that this assumption of indifference—for it could only be assumed—was pleasing to her? She had heard younger men talk in the same strain ere now, and had put them down as loose-tongued, ill-mannered boys. But to hear George Bairdsley speak thus made her heart sink; it was well that she knew him thoroughly, she told herself; had he spoken in such a way at an earlier stage of their acquaintance ——. But there! He was weak and ill, and only a week ago was raving in delirium; she must not judge him now any more than she had done when the fever made him nervous and irritable; but she was devoutly glad that there was no one within ear-shot to overhear him this evening.

"You must write to me at least twice a week, dear," she said, anxious to turn the conversation into another channel.

"It will be no task to write to you, Kitty."

"You must write such letters that I shall feel as though you were speaking to me, George; tell me your thoughts and feelings, so that I can be with you as I read."

Bairdsley promised just as Mrs. Brent's arrival put an end to their tête-a-tête, and he went slowly to his room to get ready for dinner. He felt very weak and unsteady, and earnestly wished that the long journey to Simla was over.

"Six hours' train," he said to himself, "that I don't mind. Travelling in this country is slower, but twice as luxurious as in England. Then there's the thirty-eight miles or so in a dâk-gharry from Umballa to Kalka. If the dâk-gharry is no better than the wretched wheeled kennels here, I shall be a dead man by the time I get out of it. Thereafter a drive up-hill for fifty or sixty miles in a tonga, which will shake any life I may carry so far out of me. Upon my word, were it not for Kate I would have faced the voyage and gone home."

However, the die had been cast, and the following evening saw him set out. Kate and Dr. Harrison accompanied him to the station, where they were met by a Eurasian "hospital assistant," who was to go with the invalid to Simla, acting in the joint capacity of nurse and courier. It was absolutely necessary that someone should go to look after his baggage, see to his comfort at the hotels, and engage gharries at Umballa. Kate had made the commissariat her special care, and he was as well provided for as though he were going to the end of the line at Peshawur, instead of a six hours' journey—boxes of sandwiches, and carefully packed milk puddings compounded under Mrs. Brent's housewifely eye, a huge case of ice, in which soda water had been stowed. Everything he could want had been thought of, and a great deal that he did not, besides. Mr. Da Silva, the apothecary, had a sumptuous feast next day after he had seen Captain Bairdsley to bed in the hotel.

They secured a carriage for the invalid and his attendant; a large roomy compartment, with seats running "fore and aft" under the windows, and a lavatory attached. Kate tried the revolving kuskus tattie, which stood in place of glass over the couch George occupied, saw that it would turn properly, and the little tank through which its lower half passed was full of water.

"You won't suffer from the dust now, dear, after the rain. You ought to have a comfortable run up to Umballa."

Bairdsley, whose clothes hung upon him as rags upon a scarecrow, assented, with a nod. He had driven up to the station, and even that short journey had wearied him and his one anxiety now was to turn in and sleep. The hospital assistant had been called out of the carriage to receive some last instructions from Dr. Harrison, and Bairdsley rose with an effort and took Kate in his arms.

"Good-bye, Kitty, you will wait for me? Only a few months?"

"For ever, George."

She stood at her father's side, as the train, with its odorous cargo of native passengers, glided slowly out of the station, repeating his farewell and her reply to herself.

"You will wait for me? Only a few months."

"For ever, George."

*(To be continued.)*

